# Evaluation of the Cytotoxic Effects of CAM Therapies: An *In Vitro* Study in Normal Kidney Cell Lines

**DOI:** 10.1155/2014/452892

**Published:** 2014-02-03

**Authors:** Shagun Arora, Chanderdeep Tandon, Simran Tandon

**Affiliations:** Department of Biotechnology and Bioinformatics, Jaypee University of Information Technology, Waknaghat Solan 173234, India

## Abstract

The purpose of this current study was to justify the incorporation of complementary and alternate medicine (CAM) in current cancer treatments. The major drawback of anticancer drugs is their nonselective killing, which ultimately leads to attrition of normal cells. Keeping this as the foundation of our study, we made an effort to compare the cytotoxicity associated with a known chemotherapeutic drug 5-Fluorouracil (5-FU), with certain CAM therapies previously reported to have anticancer activity. The parameters chosen for the study were based on antiproliferative and cytotoxic effects on normal, kidney epithelial cells (NRK-52E). The MTT assay, colony formation assay, DNA fragmentation, and differential staining using AO/EB, following treatment with either 5-FU or CAM therapies, were performed. The CAM therapies under study were various extracts of wheatgrass, roots of *Achyranthes aspera* (AA), mushroom extracts (*Pleurotus ostreatus, Macrolepiota procera,* and *Auricularia polytricha*), and a homeopathic drug, *Ruta graveolens* (Ruta). The results showed that treatment of normal cells with the CAM therapies led to minimum cell damage in comparison to 5-FU. This evidence-based study will lead to greater acceptance of alternative therapies against cancer.

## 1. Introduction

Kidney cancer is ranked as the 11th most common cancer in both sexes and accounts for almost 2% of all cancers, with an incidence rate of 169,155 and mortality rate of 466,631 per 100,000 people worldwide [1]. 5-Fluorouracil (5-FU) is a well-established chemotherapeutic drug used against various cancers [2, 3], which kills the cancer cell by interfering with their growth. However, this killing is nonselective and not only eliminates the fast-growing cancer cells but also other fast-growing cells in the body, including hair and blood cells, owing to which it exhibits severe toxicity and undesirable side effects [4]. 5-FU has been shown to be associated with renal toxicity [5, 6]. This puts the patient at added risk when undergoing chemotherapy. The burden of renal toxicity therefore limits the dose and duration for which the patient can be exposed to the drug.

A variety of therapeutic or preventive health care practices followed worldwide, having limited scientific literature for their effectiveness, are referred to as complementary and alternative medicines (CAM). These include homeopathy, naturopathy, Chinese traditional medicine, and Indian herbal medicine [7]. Their popularity stems from their easy availability and cost effectiveness and the fact that they have been used for centuries. Over the years, several surveys have shown the potential role of various CAM therapies over conventional therapies in cancer treatment [8–10]. According to the WHO, around 80% of the world population relies upon CAM therapies for their primary health care [11]. A survey, conducted by Indian Council of Medical Research, revealed that 38% of cancer patients opted for alternative treatments before using allopathic medicines [12, 13]. The objective of our study stems from the belief that although several chemotherapeutic drugs are available in the market against cancer, they fail to discriminate between the normal healthy cells and malicious cancer cells, thereby leading to the various side effects. Therefore, CAMs, which have been around for centuries and whose therapeutic potential is known, could better serve to protect the normal cells over conventional treatments, while at the same time killing or retarding the cancer cells.

We tested the effect of several well-documented sources of CAM therapies (*Triticum aestivum* (wheatgrass), *Achyranthes aspera* roots, Mushroom species, *Pleurotus ostreatus, Macrolepiota procera *and* Auricularia polytricha,* and homeopathic drug, *Ruta graveolens*) by exploring various parameters such as cytotoxicity, morphological alterations, antiproliferative and apoptotic effects and compared them with 5-FU on normal kidney epithelial cells (NRK-52E) [14–17].

The young grass of *Triticum aestivum *is commonly known as wheatgrass. Wheatgrass extract has been shown to possess antioxidant and anticancer activity and wound healing property, inhibits oxidative damage, and strengthens the immune system by inhibiting the metabolic activation of carcinogens [18–23]. *Achyranthes aspera *(AA) is a popular folk remedy in traditional system of medicine throughout the tropical Asian countries. This plant is reported to be used as immunostimulant, antioxidant, diuretic, antipyretic, hepatoprotective, antiproliferative, anticancerous, cytotoxic, and antiinflammatory agent and also used for treatment of renal dropsy and ulcers [24, 25].

For thousands of years, various species of mushrooms have been used as an integral part of traditional Chinese medicine and have accordingly been classified as medicinal mushrooms. Medicinal properties of mushroom are well documented and include anticancer, antioxidant, antiinflammatory, antiulcers, wound healing, and activation of immune response by activating helper T cells, cytotoxic T cells, natural killer cells, and macrophages [26, 27].

*Ruta graveolens * L. commonly known as *Rue* is an herbaceous perennial, originally native to the Mediterranean region. The therapeutic uses of Ruta include the treatment of inflammatory conditions, activation of T cells, prevention of ulcers formation, and inhibition of cancer cell proliferation [28, 29].

Studies carried out by our group have shown that these CAM therapies are highly effective against cancer cell lines. We have earlier shown that the extracts of wheatgrass effectively kill breast cancer MCF7 cells [30]. The effect of *Pleurotus ostreatus, Macrolepiota procera,* and *Auricularia polytricha *extracts were also checked on breast (MCF-7), colon (COLO-205), and kidney (ACHN) cancer cell lines in our lab. and studies revealed their cytotoxic, antiproliferative, and apoptotic effects against cancer cells [31]. The evidence for the antiproliferative effects of Ruta on cancer cells was also reported recently by Arora et al. [32]. In addition, we have seen that the extract of *Achyranthes aspera *roots has the ability to induce apoptosis in cancer cells.

Although the therapeutic activities of all these CAM therapies are reported to possess anticancer activity, the question still remains whether these therapies spare healthy cells in comparison to known chemotherapeutic drug, that is, 5-FU. Our current study has therefore tried to systematically explore this aspect of CAM therapies and document for the first time its “no-side effect policy,” through scientific fact and not belief.

## 2. Material and Methods

### 2.1. Collection of Plant Material

The seeds of *Triticum aestivum* were obtained from HAU (Chaudhary Charan Singh University, Hisar). The dried roots of *Achyranthes aspera* were procured from Natural Remedies Pvt. Ltd. at Bangalore, India, while the three species of mushroom (i.e., *Auricularia polytricha, Macrolepiota procera, *and* Pleurotus ostreatus*) were procured from Directorate of Mushroom Research, Solan, Himachal Pradesh, India. The ultradiluted (10 M, 1 M, 200 C, 30 C) potencies and mother tincture (MT) of *Ruta graveolens* were procured from Dr. Reckeweg & Co, Germany.

### 2.2. Cell Line

NRK-52E cell line was used as an *in vitro *model in our study and was obtained from NCCS Pune, India. The cells were cultured in Dulbecco's modified Eagle medium (DMEM) supplemented with 1% (v/v) Penicillin-Streptomycin obtained from Gibco and 10% (v/v) FBS obtained from Sigma-Aldrich. The cells were maintained at 37°C in a 5% CO_2_ incubator. Cells at exponential stage were used for experimentation and medium was changed every 2-3 days.

### 2.3. Preparation of Plant Extracts

#### 2.3.1. Preparation of Wheatgrass Extracts

The seeds were soaked overnight. Next day, the sprouted seeds were sowed in trays containing fertile soil. The trays were monitored daily and watered as per the need. The desired length of the wheatgrass to be used in our study was of 6-7 inches, as a particular study revealed that maximum antioxidant activity was observed in a wheatgrass of this length [33]. Successive extraction procedure was followed with an increasing order of polarity of solvent used such as hexane, chloroform, methanol, and water in a Soxhlet apparatus; subsequently the following extracts were obtained: hexane wheatgrass extract (HWE), chloroform wheatgrass extract (CWE), methanol, wheatgrass extract (MWE), and aqueous wheatgrass extract (AWE). The supernatants obtained were filtered and were further concentrated in Rotary Evaporator or lyophilized as per need.

#### 2.3.2. Preparation of AA Extracts

10% (w/v) aqueous and ethanolic extract was prepared by dissolving 10 grams of dried roots of AA in 100 mL of each solvent overnight at room temperature; next day the supernatants were filtered by Whatman filter number 2. For ethanolic extract, the filtrate obtained was further concentrated in Rotary Evaporator (at 50°–60°C) under reduced pressure leaving a dark brown residue and was transferred to a petri dish and kept in an oven (50°C) until the solvent was completely evaporated and termed as EAA-ethanolic root extract of *Achyranthes aspera*. While for aqueous extract the filtrate was lyophilized and termed as AAA-aqueous root extract of *Achyranthes aspera*.

#### 2.3.3. Preparation of Mushroom Extracts

The dried mushrooms were subjected to coarse grinding in a pestle and mortar, followed by grinding in a mixer-grinder until a fine powder was achieved. 10% (w/v) aqueous extract of all three species was prepared by dissolving 10 grams of the powdered mushroom sample in 100 mL of water, followed by extraction using Soxhlet apparatus. This process was carried out for 2-3 days until the appearance of a coloured solution and all the extracts were then lyophilized in a lyophilizer to obtain the dry powdered extract, namely, *Pleurotus ostreatus* aqueous extract (PAE), *Auricularia polytricha *aqueous extract (AAE) and * Macrolepiota procera *aqueous extract (MAE).

All the extracts were stored at −20°C until further use. The extracts which were added to the cells in culture were solubilised in cell culture grade DMSO and filtered through a 0.22 micron filter. The further dilution to obtain a range of concentrations was done using DMEM.

#### 2.3.4. Homeopathic Drug

The prepared potencies of *Ruta graveolens* used in our study were 10 M, 1 M, 200 C, 30 C, and mother tincture (MT).

### 2.4. Cytotoxicity Assessment by MTT Assay

The cytotoxic activity was measured using MTT (3-(4,5 dimethylthiazol-2-yl)-2,5-diphenyltetrazolium bromide) assay [34]. Briefly, 10^4^ cells/well were seeded in 96-well microplates. Cells were treated with various concentrations (20–120 *μ*g/mL) of HWE, CWE, MWE, AWE, PAE, MAE, AAE, EAA, and AAA and all potencies of Ruta including 10 M, 1 M, 200 C, 30 C, MT and 5-FU (1–5 *μ*g/mL) for 48 hours. At the end of incubation period, the medium was removed and 15 *μ*L MTT (5 mg/mL) was added to each well, followed by an incubation period for a further 4 hours at 37°C. Later, 150 *μ*L of DMSO was added to each well for solubilisation of the formazan products. Absorbance was taken at 570 nm using a Bio-Rad microplate reader. The percent cell cytoxicity was calculated by means of the formula:

 %  Cytotoxicity
 
$=\frac{\text{O}.\text{D}\;\text{of}\;\text{control}\;\text{sample}-\text{O}.\text{D}\;\text{of}\;\text{treated}\;\text{sample}}{\text{O}.\text{D}\;\text{of}\;\text{control}\;\text{sample}}$
 ×100.

### 2.5. Assessment of Morphological Alterations

10^5^ cells/mL were seeded in a 12-well plate and, upon reaching a confluency of 80%, were subjected to various treatments for a period of 48 hours, with IC_50_ concentrations of HWE, CWE, MWE, AWE, PAE, MAE, AAE, EAA, and AAA and 5-FU. This IC_50_ value refers to the concentration of the extract which results in 50% killing on the cells upon treatment. After 48 hours, cells were visualized to assess the changes in cell morphology and cell density in presence or absence of various CAM therapies or 5-FU and photographed under a phase contrast microscope at 100x magnification using Nikon eclipse Ti fluorescence microscope [34].

### 2.6. Antiproliferation Assay

Antiproliferative activity was investigated by performing colony forming assay (CFU) [35]; briefly 5000 cells were cultured in a 6-well plate and incubated for 48 hours with IC_50_ value of HWE, CWE, MWE, AWE, PAE, MAE, AAE, EAA, AAA and 5-FU. Ruta was added at a concentration of 5 *μ*L/mL for 48 hours. After the treatment period, old medium was replaced with fresh medium (minus any CAM or 5-FU) and incubated at 37°C for a further 10 days. Cells were then fixed with 4% paraformaldehyde for 30 minutes at 4°C and then stained with 0.5% of crystal violet for 30 minutes and counted using a colony counter.

### 2.7. Apoptosis Assay

#### 2.7.1. Acridine Orange/Ethidium Bromide Staining

Nuclear morphology of the cells was visualized by fluorescence microscopy after staining with acridine orange/ethidium bromide (AO/EB) double dye. The cells (10^5^ cells/mL) were cultured in a 12-well plate, followed by treatment with its IC_50_ value of HWE, CWE, MWE, AWE, PAE, MAE, AAE, EAA, AAA and 5-FU and 30 C and MT of Ruta (5 *μ*L/mL) for 48 hours. Later, both adherent and cells in suspension were collected and centrifuged at 130 ×g for 5 minutes. The pellet was resuspended in a solution of 25 *μ*L PBS and 2 *μ*L AO/EB dye (100 *μ*g/mL). Slides were prepared and fluorescence was observed with the help of a Nikon eclipse Ti fluorescence microscope at 200x magnification [36].

#### 2.7.2. DNA Fragmentation Assay

2 × 10^6^ cells were seeded in 35 mm tissue culture dish and incubated for 24 hours until the 80% confluency was achieved. The cells were treated with IC_50_ value of HWE, CWE, MWE, AWE, PAE, MAE, AAE, EAA, AAA and 5-FU and 30 C and MT of Ruta (5 *μ*L/mL) for 48 hours. After incubation period, both the cells in suspension and attached cells were pooled together in 1.5 mL eppendorf vial and centrifuged at 200 g for 10 minutes at 4°C. The pellet obtained was resuspended in 0.5 mL TTE (10 Mm Tris + 1 mM EDTA + 0.2% Triton ×  100) followed by vigorous vortexing and centrifuged at 20,000 ×g for 10 minutes at 4°C and the supernatant obtained upon centrifugation was collected in a new vial. To 0.5 mL supernatant, 0.1 mL ice-cold 5 M NaCl and 0.7 mL ice-cold isopropanol were added and vortexed vigorously and kept at −20°C overnight to precipitate the DNA. Next day, centrifugation at 20,000 ×g for 10 minutes at 4°C was done. The pellet obtained was washed with 0.5–0.7 mL ice-cold 70% ethanol and centrifuged at 16,100 ×g for 10 min at 4°C. Finally, the pellet was dissolved in 50 *μ*L TE (Tris- EDTA). The DNA was run on a 1% agarose gel containing ethidium bromide and visualised using a Bio-Rad Gel-Doc system [37].

### 2.8. Statistical Analysis

The results are presented as means ± SD of three independent experiments. Statistical differences among means were determined by one-way ANOVA. Differences were considered significant at *P* < 0.05. The IC_50_ values were calculated using GraphPad Prism 5.0 (GraphPad Software Inc., San Diego, CA). Every experiment included a set of negative controls (untreated cultures).

## 3. Results 

### 3.1. Evaluation of Cytotoxicity by Various CAM Therapies and 5-FU on NRK-52E Cells

**Figure 1:**
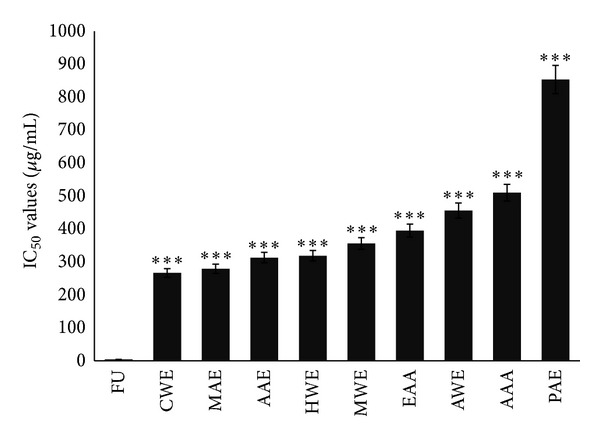
IC_50_ value of HWE, CWE, MWE, AWE, EAA, AAA, PAE, AAE, MAE, and 5-FU on NRK-52E cells measured by MTT assay after 48 hours of treatment. Data presented as mean ± S.D (*n* = 3) and compared to IC_50_ value of 5-FU. ***P < 0.001.

Normal kidney epithelial cells, NRK-52E cells, were treated with the various CAM therapies as well as 5-FU for 48 hours, to check for their cytotoxic effect. The outcome of this study revealed that, in contrast to the known chemotherapeutic drug (5-FU), the CAM therapies were significantly less toxic to normal cells. 5-FU, as indicated in Figure 1, showed remarkably stronger cytotoxic activity on the normal kidney cells with an IC_50_ value of 4.56 ± 0.41 *μ*g/mL, which was significantly lower than that of the CAM extracts under study with a IC_50_ value of 318.92 ± 0.13 *μ*g/mL for HWE, 266.67 ± 0.38 *μ*g/mL for CWE, 356.09 ± 0.26 *μ*g/mL of MWE, 456.15 ± 0.47 *μ*g/mL of AWE, 395.35 ± 0.67 *μ*g/mL of EAA, 509.83 ± 0.17 *μ*g/mL of AAA, 853.40 ± 0.51 *μ*g/mL of PAE, 313.25 ± 0.46 *μ*g/mL of AAE, and 279.12 ± 0.34 *μ*g/mL of MAE. The mother tincture (MT) of Ruta showed 19.53 ± 0.25% killing on NRK-52E cells, so IC_50_ value of Ruta on NRK-52 E cells was not calculated as 50% cell killing does not lie within the range of the ultradiluted remedies (10 M-MT) used. Further experiments were therefore done using 30 C and MT potencies. As is evident the IC_50_ values of the CAM therapies on the normal cells are very high, indicating their noncytotoxic nature.

### 3.2. Assessment of Morphological Variations of NRK-52E Cells

**Figure 2:**
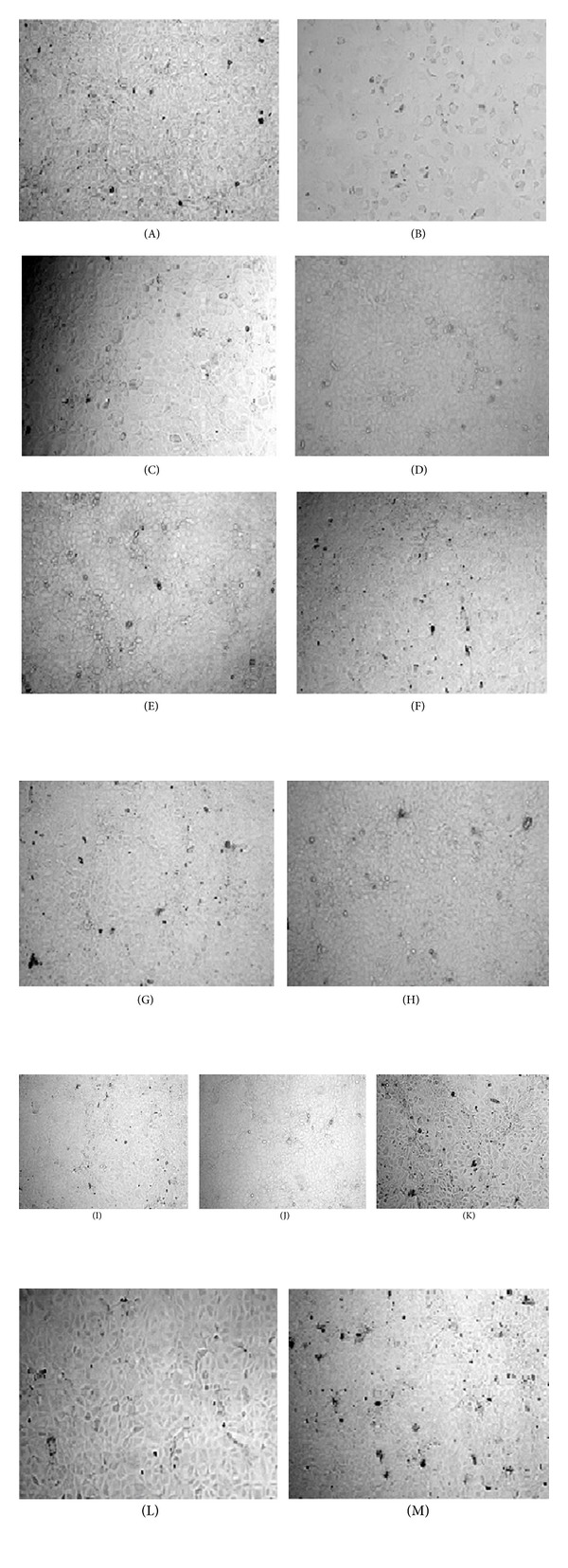
Effect of IC_50_ (based on MTT assay) values of HWE, CWE, MWE, AWE, EAA, AAA, PAE, AAE, MAE, 30 C, and MT of Ruta and 5-FU on cell growth of NRK-52E cells after treatment period of 48 hours. (A) Control (untreated); (B) 5-FU; (C) HWE; (D) CWE; (E) MWE; (F) AWE; (G) EAA; (H) AAA; (I) PAE; (J) AAE; (K) MAE; (L) 30 C; (M) MT after 48 hours treatment (magnification at 100x).

The IC_50_ values of all samples that is, HWE, CWE, MWE, AWE, PAE, MAE, AAE, EAA, AAA, 30 C, and MT potencies of Ruta, along with 5-FU, were then used for the treatment of NRK-52E cells, in a 12-well plate format, for 48 hours. Following this, the change in morphology and cell growth was evaluated and photographed as shown in Figure 2. The results revealed that the CAM therapies had hardly any growth retarding effect or any apparent effect on the morphology of the cells. In contrast the normal cells treated with 5-FU were severely affected and resulted in altered shape as well as a visible decrease in cell growth, as evidenced by clear patches seen in the wells of the plate.

### 3.3. Effect of CAM Source and 5-FU on NRK-52E Cell Proliferation

**Figure 3:**
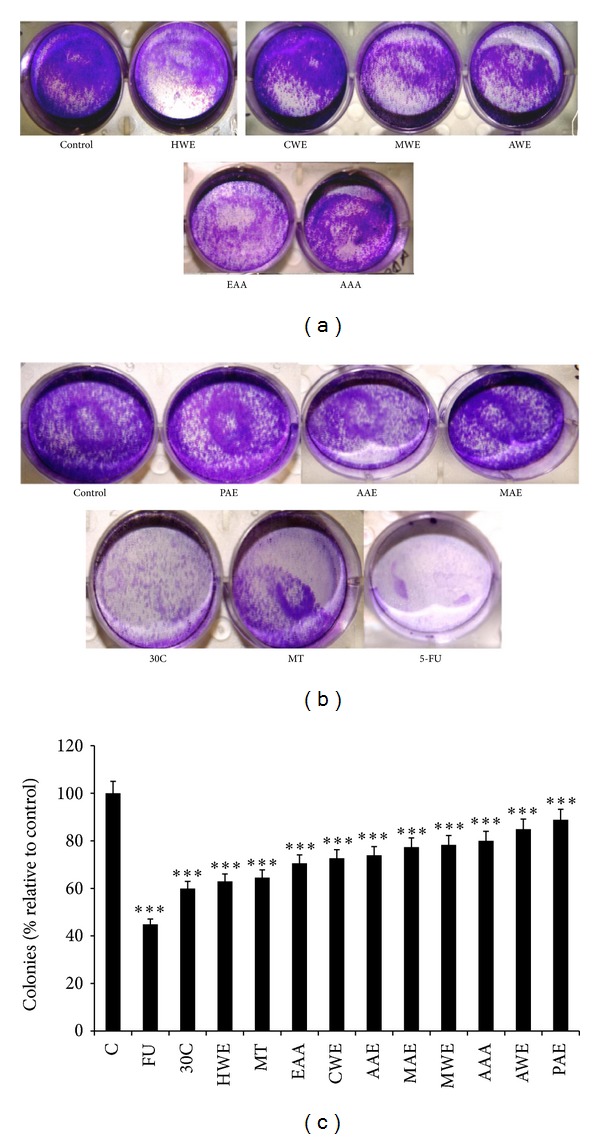
The pictographic representation of antiproliferative effect by colony formation assay of NRK-52E cells on treatment with IC_50_ values of (a) control (untreated), HWE, CWE, MWE, AWE, EAA, and AAA and (b) control (untreated), PAE, AAE, MAE, 30 C, and MT of Ruta and 5-FU for 14 days and (c) represents % percent colonies formation wrt to control (untreated).

Colony formation assay was adopted in our study as a means to evaluate the long term effect on proliferation of NRK-52E, of the various CAM treatments, in comparison to 5-FU. After an initial treatment period of 48 hours, the cells were allowed to proliferate in absence of any treatment for a further 10 days, to check the ability of cells to resume the normal cell cycle leading to proliferation. In Figure 3, a significant decrease in cell proliferation was observed, when cells were treated with 5-FU and were allowed to proliferate for the next 10 days in absence of drug, while the effect of CAM therapies on NRK-52E cells was insignificant in comparison to 5-FU.

### 3.4. Apoptotic Effect on NRK-52E Cells

#### 3.4.1. Acridine Orange/Ethidium Bromide Dual Staining

**Figure 4:**
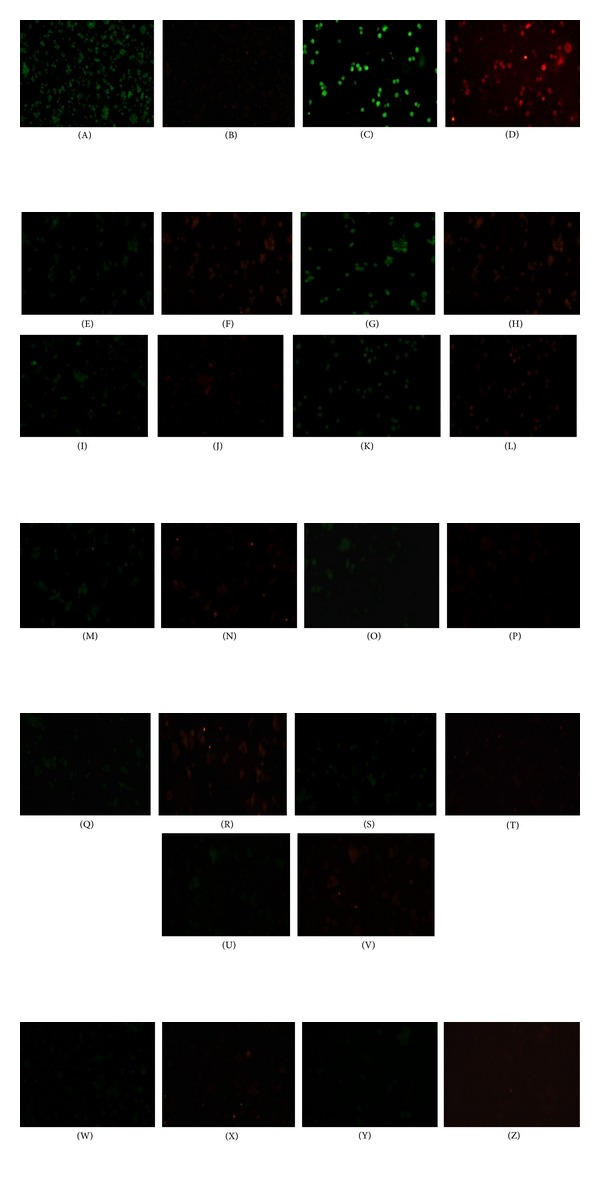
Evaluation of apoptosis in NRK-52E cells by AO/EB staining after 48 hours of treatment with IC_50_ values of HWE, CWE, MWE, AWE, EAA, AAA, PAE, AAE, MAE, 30 C, and MT of Ruta and 5-FU. Cells were observed under fluorescence microscope at 200x. (A) Control (untreated); (C) 5-FU; (E) HWE; (G) CWE; (I) MWE; (K) AWE; (M) EAA; (O) AAA; (Q) PAE; (S) AAE; (U) MAE; (W) 30 C; (Y) MT were observed under acridine orange filter and (B) control (untreated); (D) 5-FU; (F) HWE; (H) CWE; (J) MWE; (L) AWE; (N) EAA; (P) AAA; (R) PAE; (T) AAE; (V) MAE; (X) 30 C (Z) MT were observed under ethidium bromide filter.

In general, upon treatment with AO/EB dyes, the viable cells show round and green nuclei, while early apoptotic cells have fragmented DNA with green colored nuclei and late apoptotic and necrotic cells show fragmented DNA with orange and red nuclei. From the data obtained from our study, shown in Figure 4, it was clear that CAM therapies showed intact DNA and round and green nuclei, whereas 5-FU showed fragmented DNA with green colored nuclei along with typical characteristics of apoptotic cells, including plasma membrane blebbing.

#### 3.4.2. DNA Fragmentation Assay

**Figure 5:**
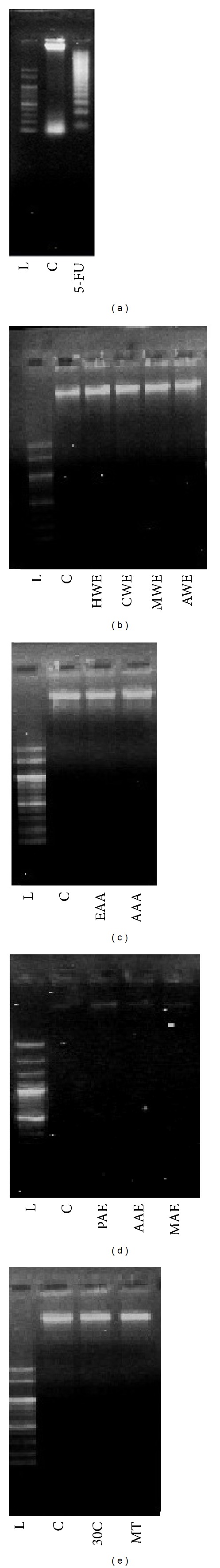
DNA fragmentation analysis in NRK-52 cells, after 48 hours of treatment with IC_50_ values of HWE, CWE, MWE, AWE, EAA, AAA, PAE, AAE, MAE, 30 C, and MT of Ruta and 5-FU. (a) L, C, 5-FU, (b) L, C, HWE, CWE, MWE, and AWE, (c) L, C, EAA, and AAA, (d) L, C, PAE, AAE, and MAE (E) L, C, 30 C, and MT. L-Ladder, C-untreated NRK-52E cells.

The process of DNA fragmentation is a clear sign of activation of apoptosis, or programmed cell death, via intrinsic pathway, which is linked to the involvement of a specific enzyme, that is, caspase activated deoxyribonuclease (CAD). CAD, upon activation by a variety of carcinogens, leads to the cleavage of the intact DNA to produce ladder-like DNA fragments of specific size of 180–200 bp. As a biochemical hallmark of cell death via apoptosis, DNA fragmentation was used to determine the effect of CAM therapies versus 5-FU effect on cell death (if any) of normal kidney epithelial cells. As shown in Figure 5, the treatment of NRK-52E cells with CAM therapies showed intact DNA with no signs of DNA fragments or laddering, indicating no apoptotic effect of these sources of CAM on normal kidney cells in contrast to 5-FU.

## 4. Discussion

In this study, we have provided the first report on the noncytotoxic nature on normal kidney cells, of certain plant extracts, having anticancer properties.

Most chemotherapeutics treatments used for the eradication of cancer cells also kill a diverse range of normal cell types, leading to a broad range of adverse side effects including renal toxicity [38, 39]. 5-FU belongs to a class of chemotherapeutic drugs, which although being highly effective leads to a wide range of toxicity within multiple organ systems, one of which is the kidney. A study reported that, on 5-FU administration, a significant increase in serum urea, creatinine, uric acid, cortisol, and potassium was observed, along with significant decrease in sodium and magnesium that leads to kidney injury. Increase in levels of malondialdehyde along with decreased levels of glutathione concentrations, after treatment with 5-FU, was also seen in renal tissues [5]. Similar results were observed in another study that showed 5-FU-induced nephrotoxicity in normal rats [6].

Although they induce severe cellular cytotoxicity and related side effects, many chemotherapeutic drugs continue to be used for the severe want of anticancer therapies with little or no toxicity to normal cells. Hence, there is an urgent need for new therapies that can offer hope. Alternative medicine is one such avenue which has been used worldwide since ancient times. However, owing to limited documented data that proves that CAM therapies have minimal or no toxic effect on normal cells, their use in conventional therapies has been restricted. We, therefore, decided to systematically evaluate the effect on normal kidney cells of proven CAM therapies, having antiproliferative and cytotoxic effect on cancer cells.

In our study four different sources of alternative therapies, with known anticancer properties, were evaluated; these included *Triticum aestivum, Achyranthes aspera*, certain medicinal mushrooms, and the homeopathic drug—Ruta [14–18]. They were individually tested on NRK-52E cells, which is a normal kidney epithelial cell line, to check whether these cells retain their healthy state upon treatment and compared the results with 5-FU.

The *in vitro* data from this study highlights the advantage of CAM therapies over 5-FU in terms of protective effect towards normal cells. Various parameters were assessed in our study, which included cell cytotoxicity by MTT assay, cell proliferation assay by colony formation assay, and apoptosis study by AO/EB dual staining and DNA fragmentation assay. The IC_50_ values of these CAM sources on NRK-52E cells, when compared with IC_50_ values of the same CAM sources on COLO-205, MCF-7, and ACHN cancer cells as given, previously in our published data, showed that these CAM therapies were significantly nontoxic to normal cells in contrast to 5-FU [28–30]. NRK-52E cells showed no change in their proliferation capacity on treatment with the various CAM extracts under study, when compared to 5-FU. This clearly indicates that 5-FU alters the growth kinetics of normal cells, while CAM therapy treated normal cells retain their normal proliferation, indicating no long time adverse effect on the cell cycle. The markers of apoptotic induction like chromatin condensation, membrane blebbing, and DNA laddering were seen by AO/EB dual staining and DNA fragmentation assay, when cells were treated with 5-FU; however, no signs of apoptosis were evident in NRK-52E cells after treatment with CAM therapies. This study clearly indicates the effective role of CAM therapies over 5-FU on normal kidney epithelial cells NRK-52E cells, thereby verifying their nonside effect policy through scientific experimentation.

## 5. Conclusion

The efficacy of CAM therapies as an effective and alternative means to eradicate cancer cells without unduly harming normal cells is a very attractive option. The mechanism whereby it spares the normal cells while killing the cancer cells is as yet unknown. Though in this present study the noncytotoxic nature, by means of cell culture, as an *in vitro* model, has shown that the extracts selectively spare normal cells, further *in vivo* animal assays should be carried out to confirm the safeties of these CAM therapies.
